# Field-of-View Optimized and Constrained Undistorted Single-Shot Study of Diffusion-Weighted Imaging and Intravoxel Incoherent Motion for the Uterus and Cervix in Females of Childbearing Age and Postmenopause: A Prospective Study

**DOI:** 10.2174/0115734056413130251204092738

**Published:** 2026-01-15

**Authors:** Shanghuang Xie, Xiaodan Li, Lesheng Huang, Lina Li, Jianfeng Hou, Ping Zhang, Zhixuan Song, Wanchun Zhang, Ling Luo, Jiahui Tang, Tianzhu Liu

**Affiliations:** 1 Lab of Molecular Imaging and Medical Intelligence, Department of Radiology, Longgang Central Hospital of Shenzhen, Shenzhen Clinical Medical College, Guangzhou University of Chinese Medicine, Longgang Central Hospital of Shantou University Medical College, Shenzhen 518116, China; 2 Department of Gynaecology, Guangdong Provincial Hospital of Traditional Chinese Medicine, Zhuhai 519000, China; 3 Department of Radiology, Guangdong Provincial Hospital of Traditional Chinese Medicine, Zhuhai 519000, China; 4 Department of Gynaecology, Longgang Central Hospital of Shenzhen, Shenzhen Clinical Medical College, Guangzhou University of Chinese Medicine, Longgang Central Hospital of Shantou University Medical College, Shenzhen 518116, China; 5 Clinical and Technical Support, Philips Healthcare, Guangzhou 510320, China

**Keywords:** Diffusion-Weighted Imaging and Intravoxel Incoherent Motion (DWI-IVIM), Uterus, Cervix, Childbearing age, Postmenopausal period, Menstrual cycle

## Abstract

**Introduction:**

The female reproductive system undergoes hormone-driven cyclical changes during the menstrual cycle and further alterations after menopause. However, the impact of these changes on imaging parameters remains unclear. This study aimed to compare diffusion-weighted imaging and intravoxel incoherent motion (DWI-IVIM) parameters of the uterus and cervix in women of childbearing age across different menstrual phases and in postmenopausal women.

**Methods:**

This prospective study was conducted from January 2022 to January 2024. Routine pelvic MRI and DWI-IVIM scans were performed in women of childbearing age during the menstrual phase (MP), follicular phase (FP), and luteal phase (LP), and at any time in postmenopausal women. Intraclass correlation coefficients (ICCs) were used to assess intra- and inter-observer repeatability of DWI-IVIM parameters.

**Results:**

Repeatability analysis demonstrated excellent intra- and inter-observer agreement for ADC, good agreement for D and f, and poor agreement for D* values. Compared with postmenopausal women, endometrial ADC, D, and D* values were significantly lower during the MP (ADC: *p* < 0.001; D: *p* = 0.003; D*: *p* = 0.007), while f values exhibited a non-significant downward trend (*p* = 0.09). No significant differences in DWI-IVIM parameters were observed in the uterine junctional zone across menstrual phases. In the myometrium, ADC values were significantly higher during the FP compared with postmenopause (*p* = 0.006). Cervical ADC, D, and f values were significantly higher across all menstrual phases than in postmenopausal women (ADC: MP/FP/LP *p* < 0.001; D: MP/FP *p* < 0.001, LP *p* = 0.02; f: MP/FP *p* < 0.001, LP *p* = 0.003), whereas D* values were significantly lower during all menstrual phases compared with postmenopause (MP/FP *p* < 0.001, LP *p* = 0.005).

**Discussion:**

These findings indicate that DWI-IVIM parameters are sensitive to physiological variations in the uterus and cervix associated with menstrual cycles and menopausal status, highlighting the importance of considering hormonal and age-related factors in clinical interpretation of functional MRI results for the female reproductive system.

**Conclusion:**

DWI-IVIM parameters can detect physiological differences in the uterus and cervix between menstrual phases and postmenopause. These findings underscore the importance of considering hormonal and physiological variability when interpreting imaging studies of the female reproductive system. Further research is warranted to explore the diagnostic implications of these parameters.

## 1 INTRODUCTION

Diseases of the female reproductive system include cervical and endometrial cancer, which have become the fourth [1] and sixth most common cancers, respectively, in women worldwide [2]. Non-invasive screening and early diagnosis are crucial for improving the outcomes of females with diseases of the reproductive system. Functional MRI sequences, such as diffusion-weighted imaging (DWI) and intravoxel incoherent motion (IVIM), have value for the diagnosis, preoperative evaluation [3], and monitoring of treatment response [4] in diseases of the female reproductive system, including uterine [5-8] and cervical cancer [9-11].

Apparent diffusion coefficient (ADC) is a DWI-derived measurement [12], that has been validated as a non-invasive imaging biomarker for assessing organopathies and efficacy of therapeutic interventions for malignancies [13-16]. However, ADC may not fully capture the properties of biological tissue, as it reflects only the magnitude of water-molecule diffusion within that tissue [17]. In contrast, IVIM, which is based on multiple DWI measurements with varying b-values, allows simultaneous assessment of the random movement of water in biological tissues due to pure molecular diffusion and perfusion [12]. Derived parameters include the pure molecular diffusion coefficient (*D*), perfusion fraction (*f*), and perfusion-related diffusion coefficient (D*) [18]. Thus, DWI-IVIM has the potential for diagnosis and monitoring diseases of the female reproductive system, including cervical and endometrial cancer [15, 19, 20].

Unlike other tissues and organs, the female reproductive system undergoes regular cyclical changes during the menstrual cycle and exhibits morphological and functional differences between the estrogen-supported reproductive phase and the estrogen-deprived menopausal phase [21, 22]. It remains uncertain whether these variations significantly influence IVIM parameters, thereby impacting the feasibility, accuracy, and reliability of IVIM in clinical diagnostics. Our recent studies have demonstrated cyclical variations in the uterus and cervix as observed through DWI-IVIM [23, 24]; however, the differences between the reproductive phase and menopause have yet to be elucidated.

Despite previous work evaluating menstrual cycle effects on IVIM parameters, a comparison between females of childbearing age and those in postmenopause is lacking. Most existing studies have focused on pathological conditions, such as cancer, often neglecting physiological differences across life stages. This evidence gap limits the ability to establish accurate baseline values or interpret functional imaging results with an age-appropriate context.

Therefore, the present study aimed to compare DWI-IVIM parameters of the uterus and cervix between females of childbearing age, evaluated across different menstrual phases, and postmenopausal women. By identifying normal physiological variations associated with age and hormonal status, we hope to provide essential baseline data for future clinical and research applications of functional pelvic MRI.

## 2 MATERIAL AND METHODS

### 2.1 Study Design and Setting

Data for these analyses were obtained from the prospective Field-of-View Optimized and Constrained Undistorted Single-Shot (FOCUS) and IVIM study that was conducted at the Guangdong Provincial Hospital of Traditional Chinese Medicine between January 2022 and January 2024 [23, 24]. The protocol for the FOCUS study was reviewed and approved by the institutional Research Ethics Board (# ZF2022-379) [23, 24]. The FOCUS study was conducted in accordance with the principles of the Declaration of Helsinki. Written informed consent was obtained from all subjects. The study flowchart is shown in Fig. (**1**).

### 2.2 Participants and Screening

Healthy females attending our hospital between January 2022 and January 2024 were eligible for this study. Inclusion criteria were: 1) no history of gynecologic diseases, 2) not using oral contraceptives or hormone replacement therapy in the last 12 months, and for postmenopausal females: 3) aged 50 or older and 4) continuous cessation of menstruation for 12 months; for females of childbearing age: 3) aged 18-45 years, 4) regular menstrual cycle (28 ± 7 days), and 5) biphasic basal body temperature. Exclusion criteria were: 1) congenital uterine anomalies, 2) leiomyoma, 3) history of adenomyosis, 4) serum hormone levels outside the normal range, 5) pregnancy, 6) contraindications to MRI, or 7) refusal to sign the consent form [23, 24].

Subjects were considered healthy based on a color Doppler ultrasound examination (Voluson S10; GE Healthcare, MA, USA) of the reproductive system, which excluded lesions in the uterus, bilateral fallopian tubes, and ovaries [23, 24]. The assessment included evaluation of the uterus, examination of myometrial uniformity, measurement of endometrial thickness, and analysis of uterine blood flow.

Normal serum hormone levels were defined as progesterone < 2.84 nmol/L during the luteal phase (LP) and > 5.8 nmol/L during the mid-luteal phase [23, 24]. Progesterone levels that did not meet the criteria for normal according to the menstrual cycle phase were retested in the next cycle. Subjects were excluded if progesterone levels remained outside the normal range.

### 2.3 Image Acquisition

Females of childbearing age underwent multiple MRIs at several time points during their menstrual cycle, including the menstrual phase (MP), defined as any of Day 1-4 of the menstrual cycle; the FP, defined as any of Day 7-12 of the menstrual cycle; and the LP, defined as any of Day 16-24 of the menstrual cycle. The ovulation phase (OP) was not included as it was too short. These timepoints were selected to capture key physiological variations associated with hormonal fluctuations across the menstrual cycle. Postmenopausal females underwent a single MRI without strict timing constraints.

MRI scans were conducted using a 3.0-T scanner (Signa Discovery 750w; GE Healthcare, MA, USA) equipped with a 16-channel abdominal coil. Bowel preparation required a low-fiber diet, fasting for 8 hours prior to the scan, and a 500 ml saline enema 30 minutes before the scan. Sequences included: 1) sagittal T2-weighted short TI inversion recovery (STIR) sequence; 2) axial T1-weighted turbo spin-echo sequence; 3) axial STIR sequence; 4) sagittal FOCUS DWI sequence; and 5) sagittal FOCUS IVIM sequence [23, 24].

The IVIM and DWI with FOCUS protocol were conducted in the sagittal plane with subjects in a supine position as previously described (Table **S1**) [23, 24]. Total scan time was 30-35 minutes.

### 2.4 Image Analysis

Image analysis was performed with the FuncTool (GE AW4.6 Advantage, GE Healthcare, USA). IVIM parameter estimation was performed using segmented bi-exponential fitting, which has been shown to improve stability and reduce noise sensitivity in clinical imaging datasets. This method first estimates the pure molecular diffusion coefficient (*D*) using a linear least-squares fit on high b-values (b ≥ 200 s/mm^2^), where perfusion contributions are considered negligible. The estimated *D* is then fixed, and the perfusion fraction (*f*) and pseudo-diffusion coefficient (D*) are calculated using a non-linear least-squares algorithm based on the Levenberg–Marquardt method.

We adopted this approach to improve the reproducibility and robustness of IVIM parameter estimation in pelvic tissues, particularly in the presence of noise, which is commonly encountered in routine clinical MRI. The choice of this fitting strategy is also supported by its reported stability compared to full bi-exponential fitting in abdominal and gynecological applications.

The b-value distribution used in our study (0, 10, 20, 30, 50, 80, 100, 150, 200, 400, 600, and 800 s/mm^2^) was selected based on a combination of prior literature and preliminary internal testing. It provides adequate sampling of both low-b (perfusion-sensitive) and high-b (diffusion-dominant) ranges while maintaining clinically acceptable scan durations.

The mono-exponential linear fitting technique was used to generate ADCs according to the following eq. (**1**) [23-25]:


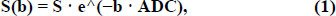


Where *S*(*b*) = mean signal intensity at a given b-value, and *S_0_* = mean signal intensity at *b*=0 s/mm^2^.

The bi-exponential model used the following eq. (**2**) [23, 24, 26]:


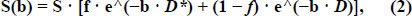


Where, *D* = pure molecular diffusion coefficient, D* = perfusion-related diffusion coefficient, *f* = perfusion fraction, and b = ≥200 s/mm^2^ and <200 s/mm^2^ [23, 24].

#### 2.4.1 ROI Placement and Standardization

Image quality was assessed by one reviewer (SX), who has over 11 years of experience in abdominal MRI. Image post-processing and parameter measurement were conducted by two radiologists (LT and HL), with 10 and 16 years of experience, respectively, in diagnosing female reproductive tract diseases on MRI. All radiologists were blinded to clinical information, including age, menstrual phase, BMI, and previous reproductive history, during ROI placement. Intra-observer repeatability of DWI-IVIM parameters was evaluated by one observer (HL) through repeated measurements conducted after an interval of at least one week.

To ensure consistency and reproducibility of ROI placement across subjects and repeated scans, all ROIs were localized using standardized anatomical landmarks on STIR images. These included the midsagittal plane of the uterus, a fixed distance from the internal os of the cervix, and a relative position within identifiable uterine layers. ROIs were consistently placed on the slice where the target structure exhibited the largest cross-sectional area, while avoiding motion artifacts and poorly defined boundaries.

Given the variability of endometrial thickness, especially in atrophic conditions, the size and shape of ROIs were adjusted based on available tissue area. In general, the ROI size was ≥5 mm^2^. If no valid ROI could be identified due to poor image quality or indistinct tissue, the subject was excluded from the endometrial analysis.

To minimize measurement errors, three non-overlapping ROIs were placed in each anatomical structure, carefully avoiding blood vessels, uterine borders, and imaging artifacts. ROI dimensions were as follows: 1) the endometrium (mean ROI area, 12 mm^2^; range, 5–20 mm^2^), anterior and posterior region of the uterine junctional zone (UJZ) (mean ROI area, 8 mm^2^; range, 5–12 mm^2^); 2) the anterior, posterior, and fundus of the myometrium (mean ROI area, 20 mm^2^; range, 15–25 mm^2^); and 3) the anterior and posterior region of the cervical muscularis (mean ROI area, 20 mm^2^; range, 15–25 mm^2^) [23, 24]. The positions of the ROIs were kept consistent across different parametric maps (Fig. **2**). Mean ADC, *D*, D*, and *f* values for each anatomical structure within the respective ROIs were calculated (Fig. **2**). Representative ROI placement is depicted in Fig. **S1**.

### 2.5 Statistical Analysis

Statistical analyses were conducted using SPSS version 26.0 (Chicago, IL, USA) and Python (v3.9) with the SciPy and statsmodels libraries. Normality testing for continuous variables used the Kolmogorov-Smirnov test. Normally distributed variables were expressed as mean ± standard deviation (SD), and non-normally distributed variables as median (minimum–maximum). Categorical variables were reported as frequencies and percentages, and analyzed with the Chi-squared test [23, 24]. Comparisons of DWI or IVIM-derived parameters between different regions (endometrium, junctional zone, myometrium, cervix) and across menstrual cycle phases (menstrual, follicular, luteal) vs. postmenopause were made using the Student’s t-test or Mann-Whitney U-test, as appropriate [23, 24]. To control for Type I errors resulting from multiple comparisons, p-values were adjusted using the Benjamini-Hochberg False Discovery Rate (FDR) procedure. A corrected *p* < 0.05 was considered statistically significant.

Intra- and inter-observer repeatability of the DWI-IVIM parameters was evaluated using intraclass correlation coefficients (ICCs), which were classified as poor (0.00–0.20), fair (0.21–0.40), moderate (0.41–0.60), good (0.61–0.80), or excellent (0.81–1.00).

All calculated ICC values, including those below 0.40, were reported along with their 95% confidence intervals, in order to provide a comprehensive evaluation of parameter repeatability. Parameters with ICC < 0.40 were considered to have limited repeatability [23].

## 3 RESULTS

### 3.1 Study Subjects

This study enrolled 60 females of childbearing age (aged 21–41 years) and 58 postmenopausal females (aged 50–70 years). Ten females of childbearing age were unable to complete all imaging sessions; 14 females (2 of childbearing age and 12 postmenopausal) were excluded due to low image quality during screening; and 13 females (2 of childbearing age and 11 postmenopausal) were excluded for intestinal gas artifacts. Finally, 46 females of childbearing age (mean age, 29.15 ± 5.10 years) and 35 postmenopausal females (mean age, 55.28 ± 4.63 years) were included in the analysis.

The characteristics of the subjects included in the final analysis are summarized in Table **1**. Among the females of childbearing age, the mean body mass index (BMI) was 20.23 ± 2.38 kg/m2, the mean menstrual cycle length was 29.76 ± 2.65 days (range: 26 – 35 days), and 20 subjects had given birth. Among postmenopausal females, the mean BMI was 22.77±5.54 kg/m2, and 33 subjects had given birth. None of the subjects included in the final analysis was obese.

### 3.2 Repeatability of DWI-IVIM Parameters

Intra- and inter-observer repeatability were excellent for ADC values (intra-observer: 0.88 and inter-observer: 0.80), good for *D* and f values (*D*: intra-observer: 0.76 and inter-observer: 0.85; *f*: intra-observer: 0.71 and inter-observer: 0.62), but poor for D* values (intra-observer: 0.07 and inter-observer: 0.04) (Table **S2**). The summarized values of the included DWI-IVIM parameters are presented in Table **2**.

### 3.3 Endometrium

ADC, *D,* and D* values for the endometrium were significantly decreased in the MP of females of childbearing age compared with postmenopause (MP versus postmenopause: ADC: *p*<0.001, *D: p*<0.001, D*: p=0.007), while *f* values showed a non-significant downward trend (*p* = 0.09). There were no significant differences between the FP and LP of females of childbearing age and postmenopause (Fig. **3** and Table **3**).

### 3.4 Uterine Junction Zone

There were no significant differences in IVIM-DWI parameter values for the UJZ (Fig. **3** and Table **3**).

### 3.5 Myometrium

The ADC value for the myometrium was significantly increased in the LP of females of childbearing age compared with postmenopause (childbearing age versus postmenopause ADC: [LP] *p* = 0.006). There were no significant differences in MP and FP between females of childbearing age and postmenopausal women (Fig. **3** and Table **3**).

### 3.6 Cervix

ADC, *D*, and *f* values for the cervix were significantly increased in the MP/LP/FP of females of childbearing age compared with postmenopause (childbearing age versus postmenopause ADC: [MP/FP/LP] *p*<0.001; *D*: [MP/FP] *p*< 0.001, [LP] *p*=0.02; *f*: [MP/FP] *p*<0.001, [LP] *p*=0.003), whereas D* values were significantly lower during all reproductive phases than in postmenopause ([MP/FP] *p* < 0.001, [LP] *p* = 0.005) (Fig. **3** and Table **3**).

## 4 DISCUSSION

This study described the DWI-IVIM parameters of the healthy uterus and cervix in females of childbearing age compared with those of postmenopausal women. Significant differences were observed in all DWI-IVIM parameters for the cervix across menstrual phases relative to postmenopause. For the endometrium, ADC, *D,* and D* values were significantly lower during MP than in postmenopause, whereas *f* values showed a non-significant decreasing trend. In the myometrium, only ADC values during the LP were significantly higher than postmenopause. No significant differences in IVIM parameters were observed for the UJZ across menstrual phases.

Research on DWI-IVIM and uterine or cervical diseases, including endometrial and cervical cancer, often overlooks the impact of the female menstrual cycle and life stages [27-30]. It is usually assumed that these factors are far less significant than the effects of the diseases and their treatments. Our results highlighted the need to raise awareness of these factors to avoid significant biases or erroneous interpretations when reviewing prior reports and conducting future research, especially in cross-sectional studies. Previously, we demonstrated that the cervix [23] and uterus [24] exhibit differences in DWI-IVIM parameters at various phases of the menstrual cycle. Our current research builds on these findings, aiming to further explore differences across various female life stages and to provide guidance for future studies investigating the female reproductive system using DWI-IVIM.

Our study demonstrated differences in DWI-IVIM parameters of the endometrium, myometrium, and cervix in females of childbearing age compared with postmenopausal women. In the endometrium, we speculate these differences are due to variations in the menstrual cycle. Our previous research indicated significant differences in DWI-IVIM parameters in the MP and HP/LP [24]. Physiological changes during the MP may impact DWI-IVIM parameters. The release of proinflammatory cytokines, chemokines, and matrix metalloproteinases causes collapse of the superficial endometrial layer, vasoconstriction of spiral arteries, and substantial constriction of the UJZ and myometrium, which may reduce uterine blood flow [22] and perfusion of the endometrium.

ADC value for the myometrium was higher across some phases of the menstrual cycle compared with postmenopause. This may be attributed to the decline in ovarian function, loss of estrogen, and atrophy of the myometrium after menopause [22]. Myometrial atrophy in postmenopausal women can be observed on conventional MRI sequences [31]. The significantly higher ADC values in the myometrium during the LP, but not the MP, in females of childbearing age, compared with postmenopause, may reflect reduced uterine peristalsis during the LP [22].

The cervix showed significant differences in all DWI-IVIM parameters between females of childbearing age and postmenopause. Evidence suggests that cervical mucosal function varies throughout the menstrual cycle [22]. The columnar epithelium, which secretes mucoprotein, responds to estradiol (E2) and progesterone. Before ovulation, elevated E2 levels increase mucus secretion and reduce its viscosity, facilitating sperm migration. After ovulation, as progesterone levels rise, mucus secretion decreases, and its viscosity increases [32, 33]. With the onset of menopause, declining ovarian function leads to reduced E2 and progesterone levels, preventing the cervix from maintaining the mucus secretion characteristic of the childbearing period [22]. Reduced E2 levels after menopause cause local atrophy and can lead to cervical stenosis, resulting in a lack of protective cervical mucus [34]. The cervical differences between childbearing and postmenopause may explain the decrease in DWI-IVIM parameters postmenopause, reflecting reduced water diffusion and decreased fluid perfusion in cervical columnar epithelial cells.

In this study, we observed that D* values in the endometrium were significantly higher during postmenopause than during the menstrual phase, and cervical D* values were consistently and markedly elevated in postmenopausal women compared with all three phases of the menstrual cycle (*p* < 0.01). These findings contrast with the trends observed for ADC, D, and f values, which were all lower after menopause.

The elevated D* values in postmenopausal tissues may reflect altered vascular architecture and tissue remodeling associated with estrogen deprivation. In the cervix, local atrophy, loss of glandular structure, and increased fibrosis may contribute to inconsistent perfusion behavior and unstable IVIM signal fitting at low b-values, leading to apparent D* inflation. Similarly, in the endometrium, absence of cyclical vasoconstriction and tissue shedding may result in reduced microvascular pulsatility but increased susceptibility to perfusion-related signal noise.

However, the poor repeatability of D* in our study (ICC: intra-observer 0.07; inter-observer 0.04) suggests that this parameter is highly sensitive to signal noise, motion artifacts, and model instability, particularly in small or atrophic tissues. This is consistent with prior literature, which has highlighted the limitations of D* in clinical use due to its low robustness and large variability [12, 17, 18].

Despite these challenges, our findings indicate that D* may carry exploratory physiological significance, especially in the context of menopausal tissue remodeling. Advanced fitting methods, such as Bayesian approaches, total variation regularization, or model-constrained algorithms, may improve D* estimation in future studies [18, 26]. Until then, D* should be interpreted cautiously and used alongside other IVIM parameters when assessing perfusion in hormone-sensitive pelvic tissues.

Aside from our research, few investigations have explored monoexponential and advanced DWI in relation to the female menstrual cycle and life stages. Some studies [35, 36] have demonstrated that the uterus undergoes periodic changes in DWI and diffusion kurtosis imaging (DKI) parameters corresponding to physiological cycles. Another study [37] found that postmenopausal women have lower regional fractional anisotropy (FA) and ADC values compared with younger women. Our research helps clarify the cyclical changes in DWI-IVIM parameters for the uterus and cervix of females of childbearing age and the differences between females of childbearing age and postmenopause. Our results will guide future DWI-IVIM research on the female reproductive system, especially for cervical cancer or other cervical disorders, suggesting that subgroup or stratified analyses may be necessary. Our comprehensive analysis of the DWI-IVIM characteristics of the normal uterus across different physiological cycles and life stages provides potential research avenues for *in vivo* physiological studies.

## 5 LIMITATION

This study has several limitations. First, this was a single-center study with a relatively small sample size. Although our sample size was determined based on feasibility and our prior research, a formal a priori power calculation was not performed due to the lack of comparable studies using IVIM-DWI in this population. We acknowledge this as a limitation and encourage future studies with larger cohorts and power analyses to validate our findings. Second, scanner drift and calibration were not assessed during the 2-year recruitment period. Although all scans were performed using the same MRI scanner and protocol, potential changes in hardware or software over time may have introduced measurement variability. Third, we excluded perimenopausal females due to the variability and uncertainty of their hormonal status and menstrual cycle characteristics. This may limit the generalizability of our results to this transitional population. Finally, despite the use of FOCUS technology and motion artifact reduction techniques, image quality was occasionally affected by bowel gas, leading to the exclusion of some subjects and potentially influencing measurement precision. As clinical imaging workflows become increasingly digitized, future studies may explore integrating our physiological DWI-IVIM benchmarks into intelligent imaging frameworks. For example, automated parameter extraction using deep learning, secure data handling via blockchain, and real-time analysis through smart IoT-based MRI systems may advance personalized gynecologic diagnostics [38-40].

## CONCLUSION

In conclusion, our findings highlight measurable differences in DWI-IVIM parameters of the uterus and cervix across various menstrual phases in childbearing age women compared to postmenopausal women. While these results enhance our understanding of physiological variability, they should be interpreted as hypothesis-generating. Future studies incorporating patient cohorts with known uterine and cervical pathologies are necessary to validate whether these physiological differences can help differentiate normal from pathological conditions and to establish clinically applicable diagnostic thresholds.

## Figures and Tables

**Fig. (1):**
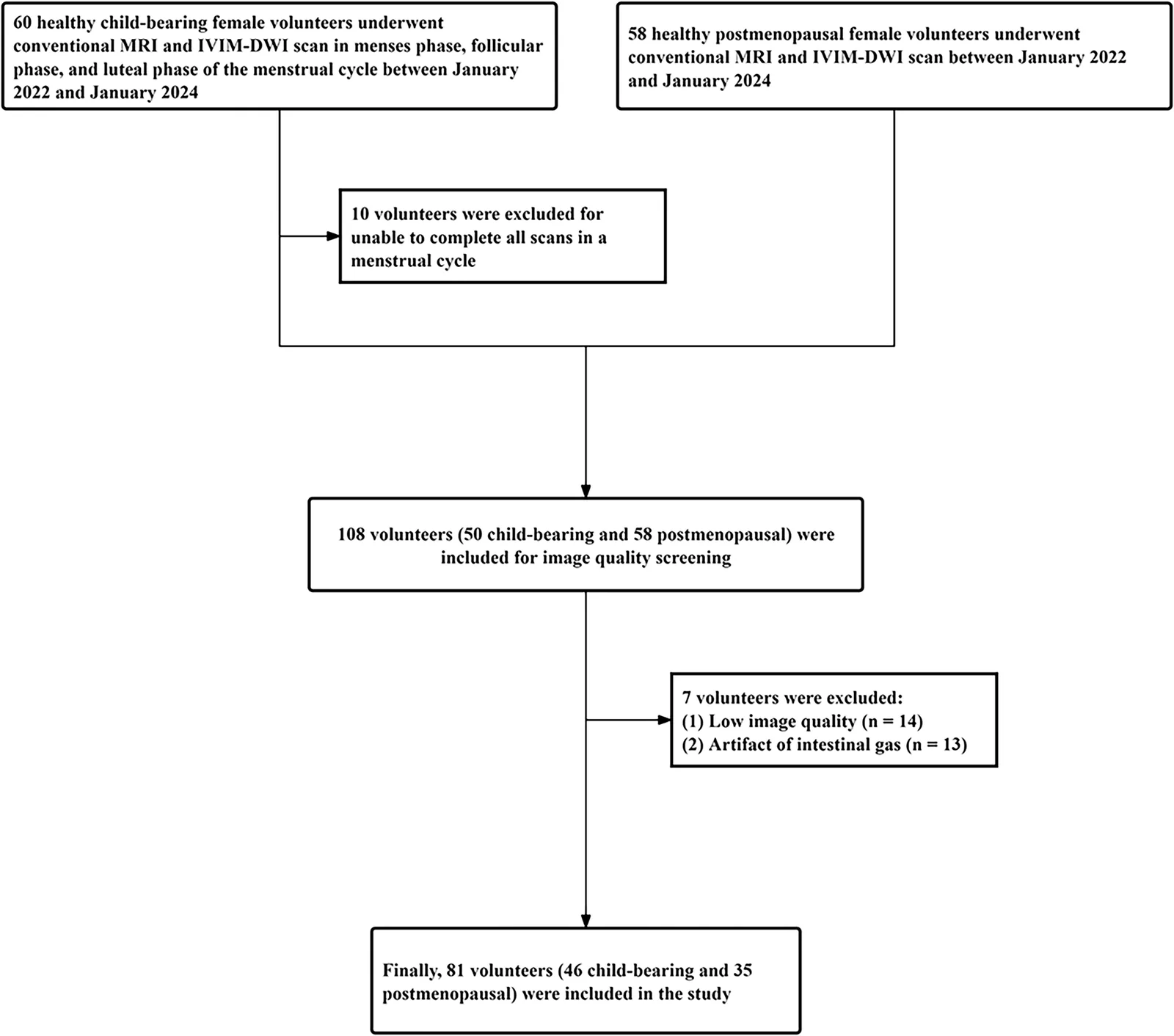
Flow chart of subject selection.

**Fig. (2):**
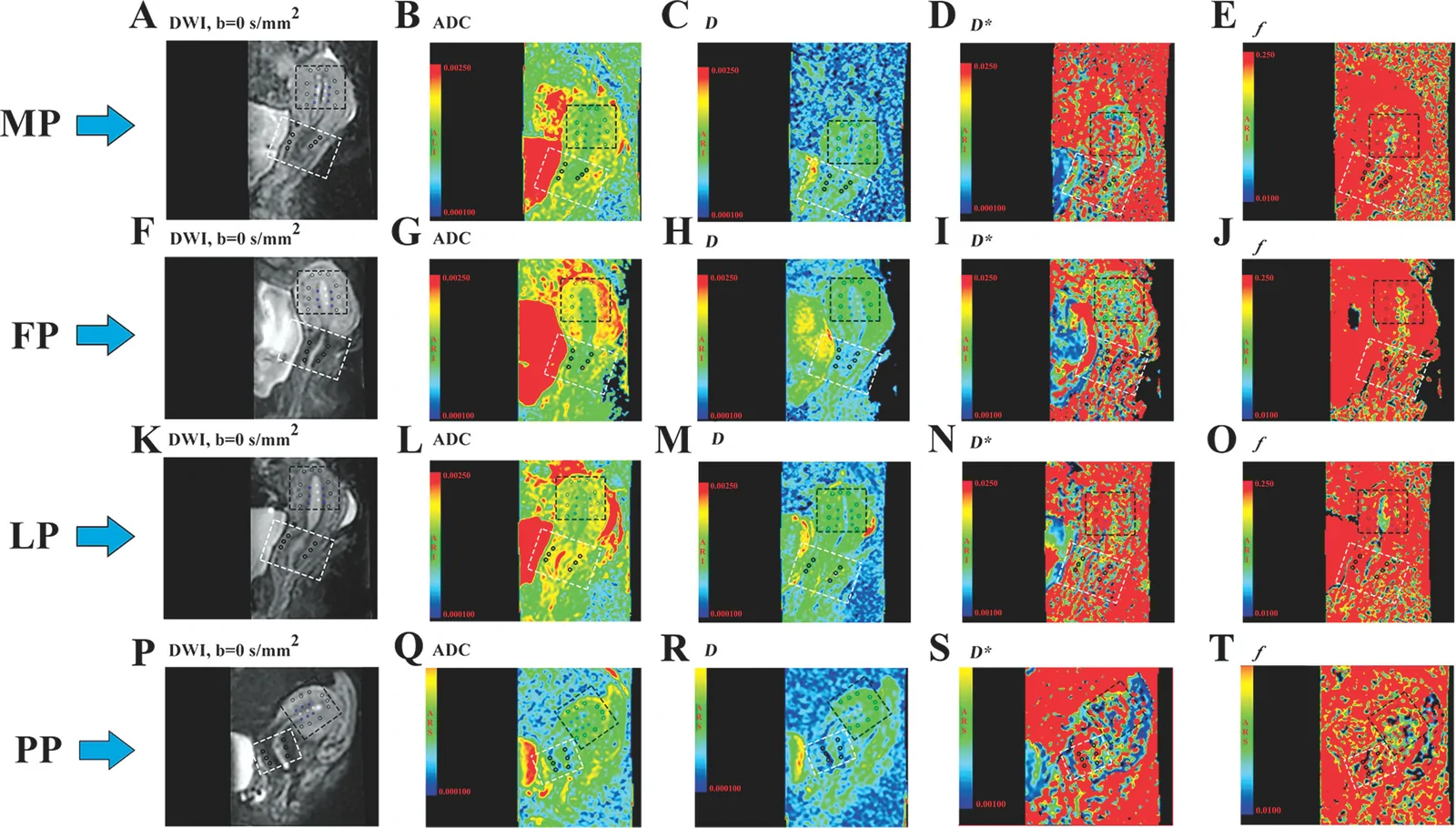
Images of a 25-year-old healthy female of childbearing age at the MP, FP, and LP (**A**-**O**), and a 53-year-old healthy postmenopausal female (P-T). (**A**, **F**, **K**, **P**: Sagittal DWI, b = 0 s/mm^2^; **B**, **G**, **L**, **Q**: ADC map; **C**, **H**, **M**, **R**: *D* values; **D**, **I**, **N**, **S**: D* values; **E**, **J**, **O**, **T**: *f* values. DWI-IVIM parameters were measured in the sagittal view. ROIs for the cervix (white dashed rectangle) were located on the central part of cervix (black thick circles, **A**-**T**); ROIs for the uterus (black dashed rectangle) were located on the central part of the endometrium (white thin circles, **A**-**T**), the UJZ (blue thin circles, **A**-**T**), and the myometrium (black thin circles, **A**-**T**).

**Fig. (3):**
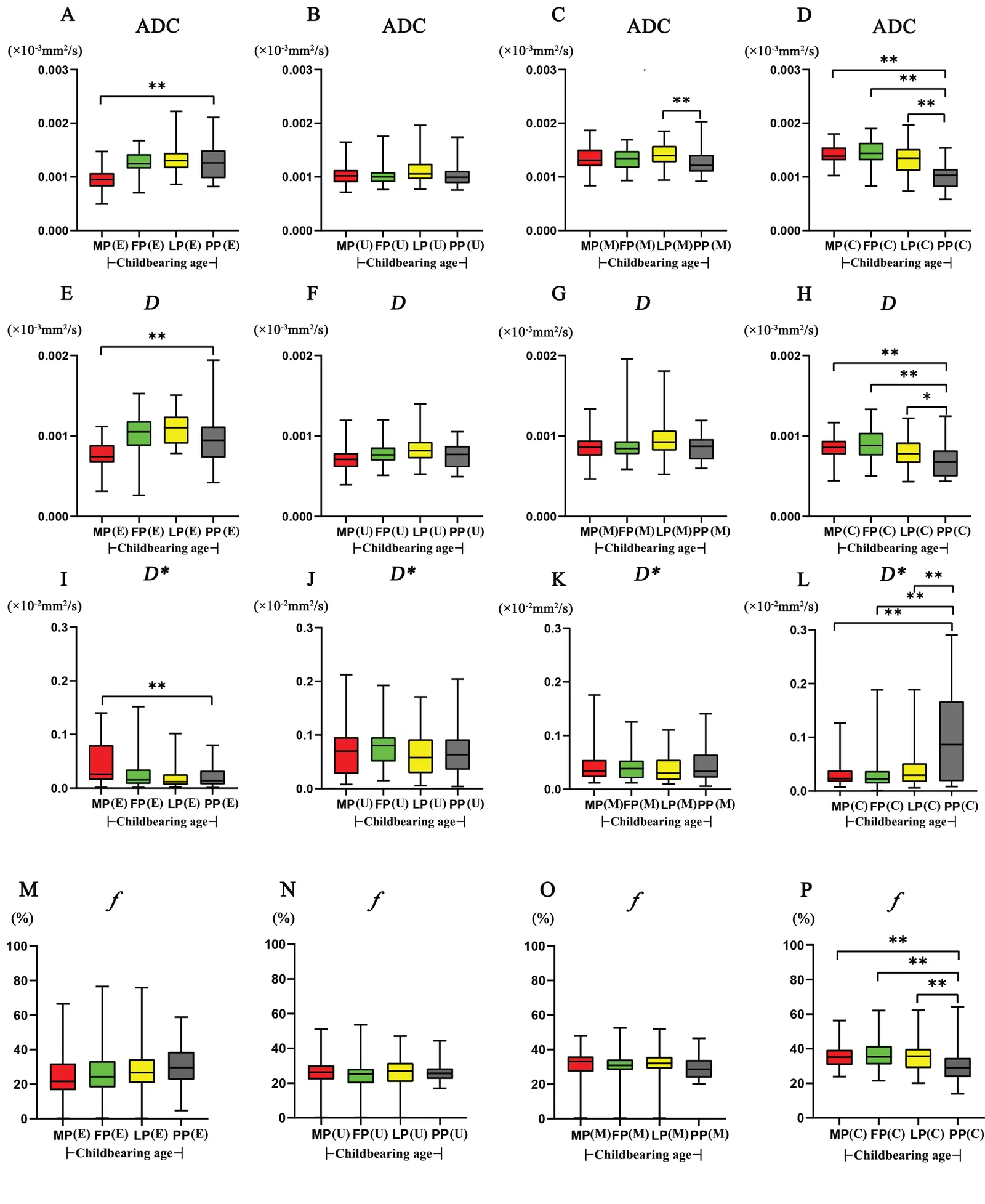
Box plots for females of childbearing age (MP, FP, and LP) and postmenopausal females (PP) of DWI-IVIM parameters for the endometrium, UJZ, myometrium, and cervix. ADC, D, D* and *f* values for the endometrium showed differences between the MP and PP (A, E, I, M); ADC and *D* values for the UJZ showed differences between the LP and PP (B, F); ADC and *D* values for the myometrium showed differences between the MP/LP and PP (C, G); ADC, D, D* and *f* values for the cervix showed differences between the MP/FP/LP and PP (D, H, L, P). (E: endometrium; U: UJZ; M: myometrium; PP: postmenopausal period; *: *p* < 0.05; **: *p* < 0.01).

**Table 1:** Baseline characteristics of the subjects included in the final analysis.

**Characteristics**	**Childbearing Age**	**Postmenopausal**
Population	46	35
Age (years)*	29.15 ± 5.10 (21–41)	55.28 ± 4.63 (50-70)
Procreation (N/O/M)	26/12/8	2/22/11
Birth model (Not yet/Cesarean/eutocia)	26/8/21	2/9/64
Menstrual cycle (days)*	29.76 ± 2.65 (26-35)	N/A
BMI (kg/m^2^)	20.23±2.38(16.53-26.67)	22.77±5.54(18.08-27.43)

**Note:** *Data presented as mean±standard deviation, and data in parentheses are the range.

N, none; O, one; M, more than one; BMI, Body Mass Index.

**Table 2:** ADC, *D* and *f* values.

**DWI-IVIM parameter**	**ADC (×10^−3^mm^2^/s)**	***D* (×10^−3^mm^2^/s)**	D* (×10^−2^mm^2^/s)	***f* (%)**
Endometrium	-	-	-	-
MP (Childbearing age, n = 46)	0.97 ± 0.20	0.76 ± 0.18	4.45 ± 3.92	25.48 ± 12.67
FP (Childbearing age, n = 46)	1.27 ± 0.19	1.03 ± 0.22	2.25 ± 2.61	27.60 ± 13.43
LP (Childbearing age, n = 46)	1.34 ± 0.23	1.10 ± 0.19	2.06 ± 2.20	29.77 ± 14.30
PP (n = 35)	1.30 ± 0.33	1.00 ± 0.32	2.28 ± 2.28	29.93 ± 11.52
Uterine Junction Zone	-	-	-	-
MP (Childbearing age, n = 46)	1.04 ± 0.20	0.72 ± 0.15	6.80 ± 4.38	27.12 ± 6.80
FP (Childbearing age, n = 46)	1.03 ± 0.19	0.77 ± 0.12	7.97 ± 4.26	25.53 ± 6.66
LP (Childbearing age, n = 46)	1.10 ± 0.20	0.83 ± 0.16	6.51 ± 4.56	26.69 ± 7.14
PP (n = 35)	1.02 ± 0.19	0.76 ± 0.15	6.97 ± 4.92	26.58 ± 6.27
Myometrium	-	-	-	-
MP (Childbearing age, n = 46)	1.35 ± 0.24	0.86 ± 0.17	4.44 ± 3.23	32.68 ± 5.90
FP (Childbearing age, n = 46)	1.33 ± 0.19	0.87 ± 0.21	4.43 ± 2.82	31.74 ± 5.70
LP (Childbearing age, n = 46)	1.41 ± 0.22	0.96 ± 0.22	3.72 ± 2.44	32.63 ± 6.24
PP (n = 35)	1.25 ± 0.22	0.85 ± 0.15	4.45 ± 3.12	29.79 ± 6.84
Cervix	-	-	-	-
MP (Childbearing age, n = 46)	1.41 ± 0.18	0.85 ± 0.15	3.07 ± 2.22	35.77 ± 7.13
FP (Childbearing age, n = 46)	1.44 ± 0.23	0.90 ± 0.19	3.35 ± 3.35	36.04 ± 8.10
LP (Childbearing age, n = 46)	1.31 ± 0.28	0.78 ± 0.17	4.38 ± 4.12	34.82 ± 8.77
PP (n = 35)	1.02 ± 0.25	0.68 ± 0.19	11.18 ± 9.71	29.30 ± 9.25

**Note:** Data are presented as mean ± standard deviation.

MP, Menstrual Phase; FP, Follicular Phase; LP, Luteal Phase; PP, Postmenopausal Period.

**Table 3:** The *p*-values for statistical comparisons between different phases of the menstrual cycle and postmenopause.

***p*-value of DWI-IVIM parameter**	**ADC**	***D***	D*	***f***
*Endometrium*	-	-	-	-
MP *vs*. PP	**< 0.001**	**0.003**	**0.007**	0.09
FP *vs*. PP	0.92	0.38	0.49	0.32
LP *vs*. PP	0.49	0.08	0.81	0.61
*Uterine junction zone*	-	-	-	-
MP *vs*. PP	0.81	0.66	0.81	0.81
FP *vs*. PP	0.81	0.81	0.53	0.81
LP *vs*. PP	0.20	0.21	0.81	0.81
*Myometrium*	-	-	-	-
MP *vs*. PP	0.24	0.90	0.57	0.20
FP *vs*. PP	0.27	0.66	0.57	0.44
LP *vs*. PP	**0.006**	0.20	0.58	0.26
*Cervix*	-	-	-	-
MP *vs*. PP	**<0.001**	**<0.001**	**<0.001**	**<0.001**
FP *vs*. PP	**<0.001**	**<0.001**	**<0.001**	**<0.001**
LP *vs*. PP	**<0.001**	**0.02**	**0.005**	**0.004**

**Note:** MP, Menstrual Phase; FP, Follicular Phase; LP, Luteal Phase; PP, Postmenopausal Period; UJZ, Uterine Junction Zone.

## Data Availability

The data of current study are available from corresponding author, [T.L], on a reasonable request.

## LIST OF ABBREVIATIONS

DWI-IVIM: Diffusion-weighted Imaging and Intravoxel Incoherent Motion
MRI: Magnetic Resonance Imaging
MP: Menstrual Phase
FP: Follicular Phase
LP: Luteal Phase
ICC: Intraclass Correlation Coefficients
ADC: Apparent Diffusion Coefficient
D*: Perfusion-related Diffusion Coefficient
D: Pure Molecular Diffusion Coefficient
f: Perfusion Fraction
FOCUS: Field-of-view Optimized and Constrained Undistorted Single-shot
OP: Ovulation Phase
ROI: Region Of Interest
UJZ: Uterine Junctional Zone
SD: Standard Deviation
BMI: Body Mass Index

## STANDARDS OF REPORTING

STROBE guidelines were followed.
