# Supplementary material for: Field-of-View Optimized and Constrained Undistorted Single-Shot Study of Diffusion-Weighted Imaging and Intravoxel Incoherent Motion for the Uterus and Cervix in Females of Childbearing Age and Postmenopause: A Prospective Study
Source: Curr Med Imaging. 2026 Jan 15;22:E15734056413130. doi: 10.2174/0115734056413130251204092738 (PMC13617554; doi:10.2174/0115734056413130251204092738)
Supplement: Supplementary file 1 [file CMIM-22-E15734056413130_SD1.pdf]

1875-6603/26 *Send Orders for Reprints to reprints@benthamscience.net*

BENTHAM SCIENCE

CrossMark

Current Medical Imaging

Content list available at: https://benthamscience.com/journals/cmir

Supplementary Material

Field-of-View Optimized and Constrained Undistorted Single-Shot Study of Diffusion-Weighted Imaging and Intravoxel Incoherent Motion for the Uterus and Cervix in Females of Childbearing Age and Postmenopause: A Prospective Study

Shanghuang Xie[1,#], Xiaodan Li[2,#], Lesheng Huang[3], Lina Li[2], Jianfeng Hou[2], Ping Zhang[4], Zhixuan Song[5], Wanchun Zhang[3], Ling Luo[1], Jiahui Tang[3] and Tianzhu Liu[3,*]

[1]*Lab of Molecular Imaging and Medical Intelligence, Department of Radiology, Longgang Central Hospital of Shenzhen, Shenzhen Clinical Medical College, Guangzhou University of Chinese Medicine, Longgang Central Hospital of Shantou University Medical College, Shenzhen518116, China*
[2]*Department of Gynaecology, Guangdong Provincial Hospital of Traditional Chinese Medicine, Zhuhai519000, China*
[3]*Department of Radiology, Guangdong Provincial Hospital of Traditional Chinese Medicine, Zhuhai519000, China*
[4]*Department of Gynaecology, Longgang Central Hospital of Shenzhen, Shenzhen Clinical Medical College, Guangzhou University of Chinese Medicine, Longgang Central Hospital of Shantou University Medical College, Shenzhen518116, China*
[5]*Clinical and Technical Support, Philips Healthcare, Guangzhou510320, China*

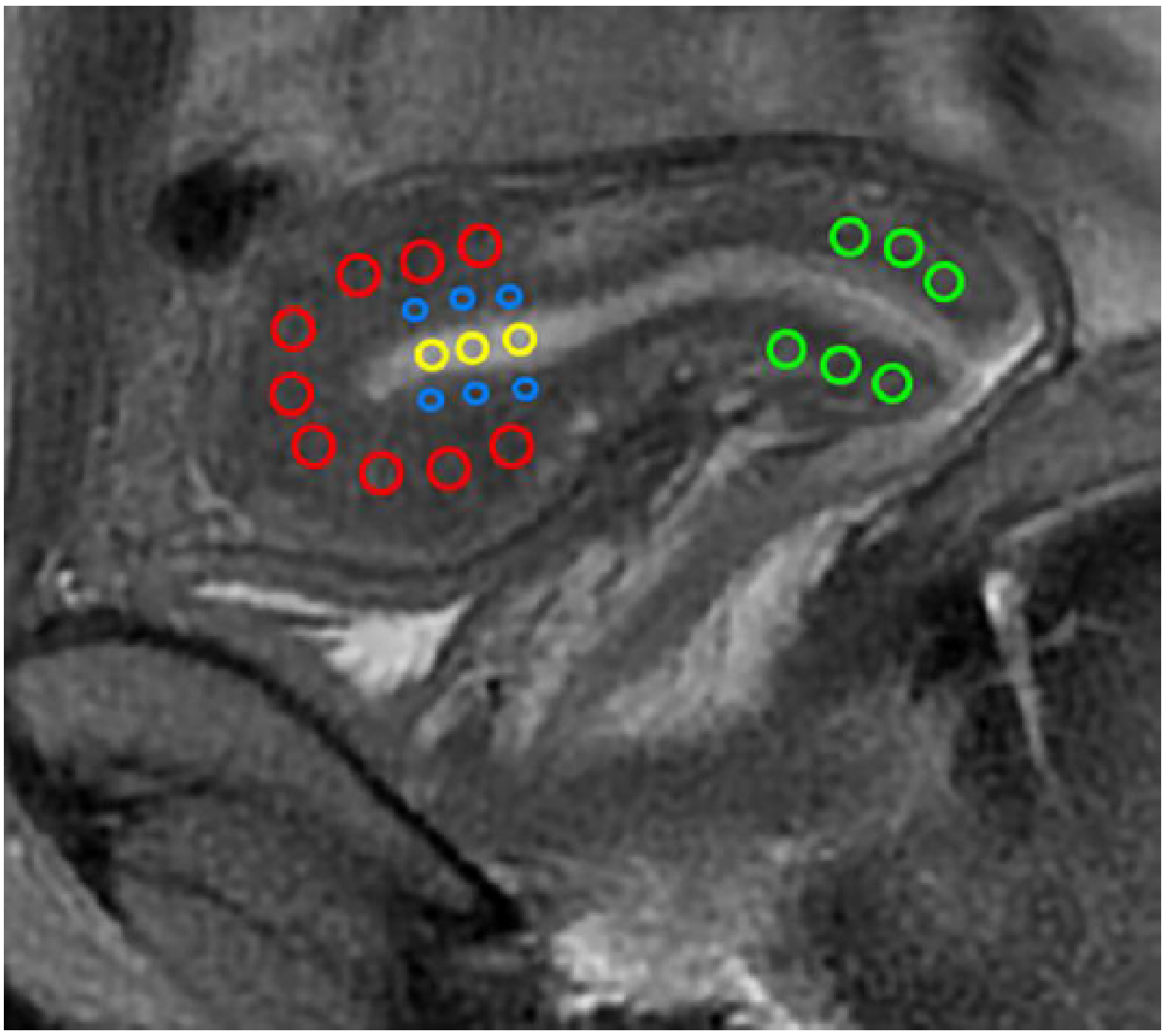

Fig. (S1). STIR image from a 31-year-old healthy female at the MP. ROIs were placed on the cervix (green circles) and on the uterus, including the central part of the endometrium (yellow circles), the UJZ (blue circles), and the myometrium (red circles).

DOI: 10.2174/0115734056413130251204092738, 2026, *22*, e15734056413130

Table S1. Magnetic resonance scanning parameters.

MRI Scan Sequence	Imaging Parameters
Sagittal T2-weighted short TI inversion recovery sequence	Average repetition time = 3000 ms
Axial T1-weighted turbo spin echo sequence	Average echo time = 69 ms
Axial STIR sequence	Slice thickness = 4 mm
Sagittal FOCUS DWI sequence (0 and 800 s/mm^2)	Inter-slice gap = 1 mm
Sagittal FOCUS IVIM sequence (0, 10, 20, 30, 50, 80, 100, 150, 200, 400, 600, and 800 s/mm^2)	Matrix = 128 × 128
-	Field-of-view (FOV) = 240 mm × 240 mm
-	Number of slices =16 – 20

Note: STIR: short TI inversion recovery; FOCUS: Field-of-View Optimized and Constrained Undistorted Single-Shot; DWI: diffusion-weighted imaging; IVIM: intravoxel incoherent motion.

Table S2. ICC of IVIM-DWI values in intra- and interobserver.

IVIM-DWI Parameters	Intra-observer (95% CI)	Inter-observer (95% CI)
ADC	0.88 (0.81 – 0.94)	0.80 (0.68 – 0.90)
D	0.76 (0.60 – 0.88)	0.85 (0.75 – 0.93)
*D**	0.07 (–0.20 – 0.35)	0.04 (–0.22 – 0.30)
f	0.71 (0.55 – 0.84)	0.62 (0.40 – 0.79)

© 2026 The Author(s). Published by Bentham Science Publisher.

This is an open access article distributed under the terms of the Creative Commons Attribution 4.0 International Public License (CC-BY 4.0), a copy of which is available at: https://creativecommons.org/licenses/by/4.0/legalcode. This license permits unrestricted use, distribution, and reproduction in any medium, provided the original author and source are credited.

HOW TO CITE:
Xie S, Li X, Huang L, Li L, Hou J, Zhang P, Song Z, Zhang W, Luo L, Tang J, Liu T. Field-of-View Optimized and Constrained Undistorted Single-Shot Study of Diffusion-Weighted Imaging and Intravoxel Incoherent Motion for the Uterus and Cervix in Females of Childbearing Age and Postmenopause: A Prospective Study. Curr Med Imaging, 2026; 22: e15734056413130.
http://dx.doi.org/10.2174/0115734056413130251204092738

DISCLAIMER: The above article has been published, as is, ahead-of-print, to provide early visibility but is not the final version. Major publication processes like copyediting, proofing, typesetting and further review are still to be done and may lead to changes in the final published version, if it is eventually published. All legal disclaimers that apply to the final published article also apply to this ahead-of-print version.
